# Supplementary material for: The nodD1 Gene of Sinorhizobium fredii HH103 Restores Nodulation Capacity on Bean in a Rhizobium tropici CIAT 899 nodD1/nodD2 Mutant, but the Secondary Symbiotic Regulators nolR, nodD2 or syrM Prevent HH103 to Nodulate with This Legume
Source: Microorganisms. 2022 Jan 10;10(1):139. doi: 10.3390/microorganisms10010139 (PMC8780172; doi:10.3390/microorganisms10010139)
Supplement: Supplementary file 1 [file microorganisms-10-00139-s001.zip › Fuentes-Romeroetal2021_Supplementary.pdf]

The *nodD1* gene of *Sinorhizobium fredii* HH103 restores nodulation capacity on bean in a *Rhizobium tropici* CIAT 899 *nodD1 nodD2* mutant, but the secondary symbiotic regulators *nolR*, *nodD2* or syrM prevent HH103 to nodulate with this legume

Francisco Fuentes-Romero, Pilar Navarro-Gómez, Paula Ayala-García, Isamar Moyano-Bravo, Francisco-Javier López-Baena, Francisco Pérez-Montaño, Francisco-Javier Ollero-Márquez, Sebastián Acosta-Jurado *, José María Vinardell González,*

List of Supplementary Material

Figure S1. Nodule primordia (panel A) and lateral roots (panel B) on *Phaseolus vulgaris* roots. Photos on the left were taken under a Leica microscope (100x). Photos on the right were taken under a binocular loupe (bars correspond to 0.5 cm).

Figure S2. Symbiotic performance of *R. tropici* CIAT 899, *S. fredii* HH103 and its *nodD2*, *nolR*, *syrM*, and *ttsI* derivatives with *Phaseolus vulgaris*. Appearance of roots.

Table S1. Bacterial strains and plasmids used in this work.

Dataset S1. List and areas of Nod factors produced by *R. tropici* CIAT 899, CIAT 899 Δ*nodD1D2*, and CIAT 899 Δ*nodD1D2* (pMUS296) upon induction with apigenin. Nod factors produced by CIAT 899 in the absence of flavonoids are also shown.

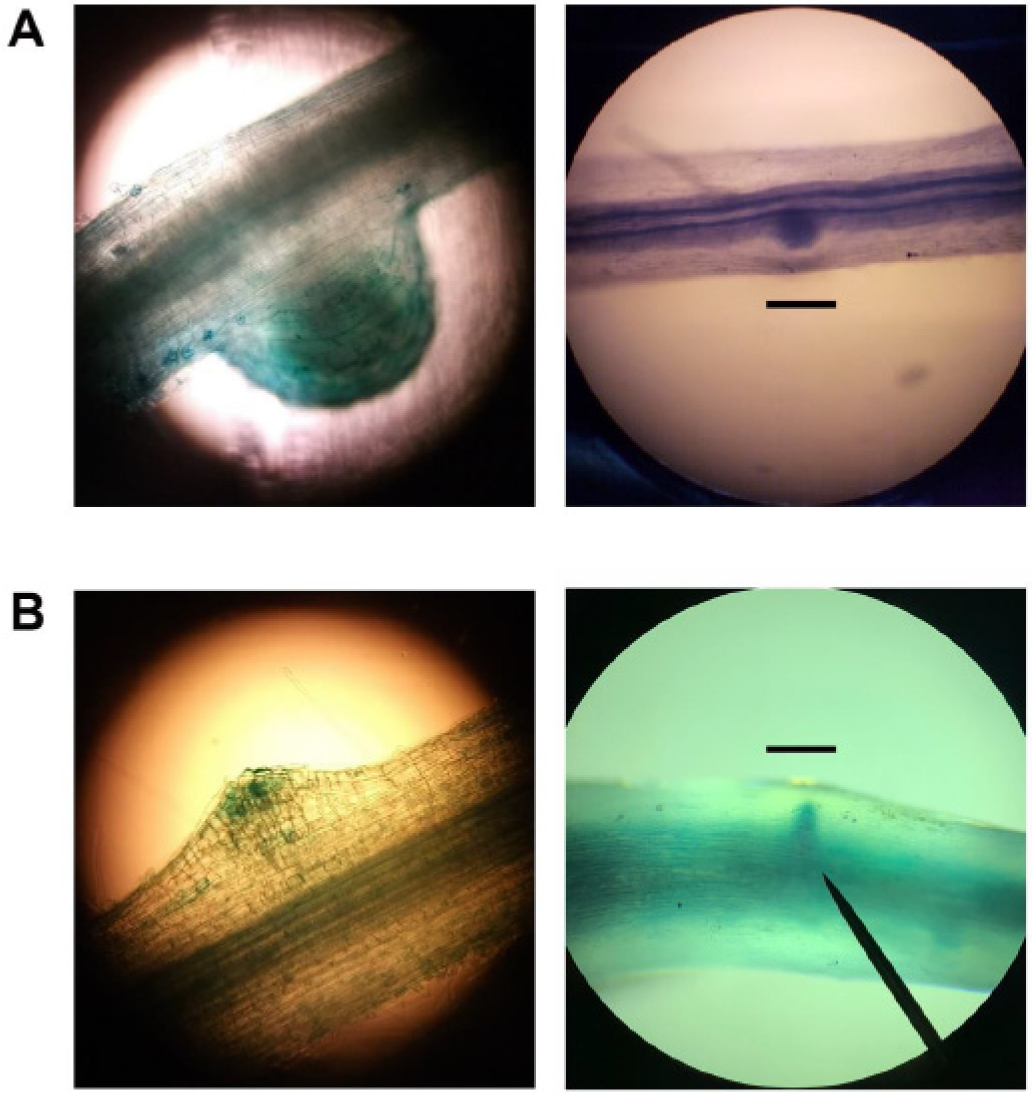

Figure S1. Nodule primordia (panel A) and lateral roots (panel B) on *Phaseolus vulgaris* roots. Photos on the left were taken under a Leica BioMed (Germany) microscope (100x). Photos on the right were taken under a Optika SZN 4 (Italy) binocular loupe (bars correspond to 0.5 cm).

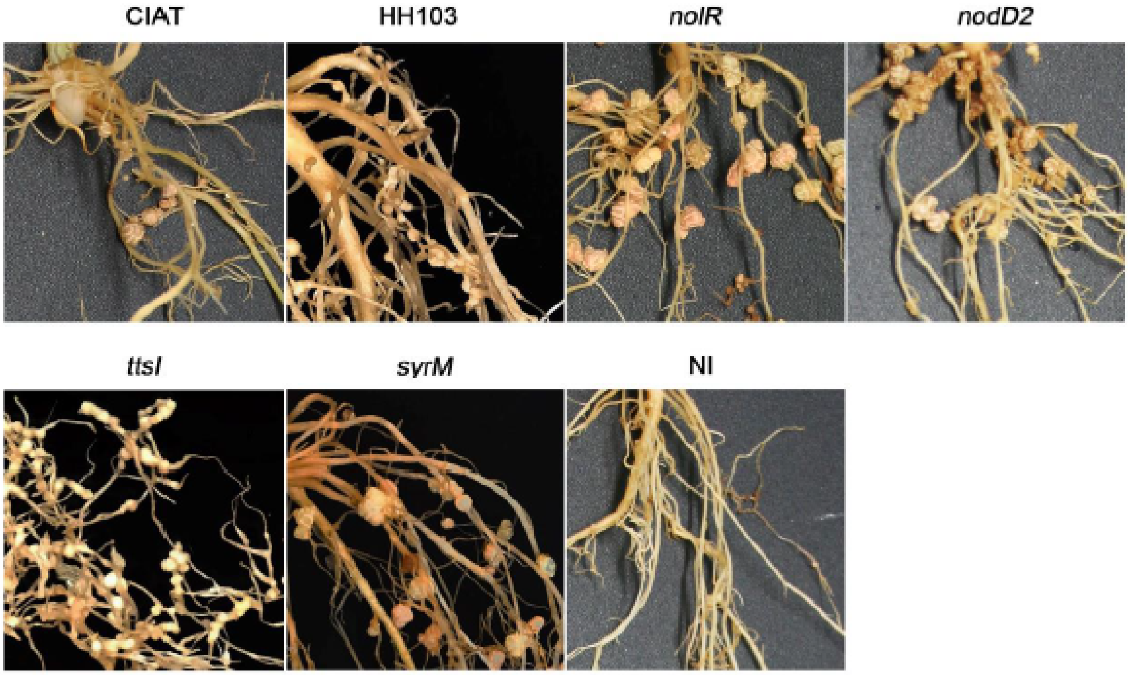

Figure S2. Symbiotic performance of *R. tropici* CIAT 899, *S. fredii* HH103 and its *nodD2*, *nolR*, *syrM*, and *ttsI* derivatives with *Phaseolus vulgaris*. Appearance of roots.

Table S1. Bacterial strains and plasmids used in this work.

Strain	Derivation and relevant properties[a]	Source or reference
Rhizobium tropici		
CIAT 899	Wild-type strain (Rif_R)	[1]
CIAT 899 Δ*nodD1* Δ*nodD2*	CIAT 899 Δ*nodD1* Δ*nodD2*	[2]
Sinorhizobium fredii		
HH103 RifR	Spontaneous Rif_R derivative of HH103	[3]
HH103 Rif_R *nodD2*	HH103 RifR nodD2::	[4]
HH103 RifR nolR	HH103 Rif_R *nolR::lacZ*Δp-GmR	[5]
HH103 Rif_R *syrM*	HH103 Rif_R ΔsyrM	[6]
HH103 Rif_R *ttsI*	HH103 RifR ttsI::	[4]
Plasmids		
pMUS296	Broad host range plasmid pMP92 carrying HH103 *nodD1*, Tc_R	[7]
pMUS746	pMP92 carrying HH103 *nodD2*, Tc_R	[4]
pRK2013	Helper plasmid, KmR	[8]

References

1. Martínez-Romero, E.; Segovia, L.; Mercante, F.M.; Franco, A.A.; Graham, P.; Pardo, M.A. *Rhizobium tropici*, a novel species nodulating *Phaseolus vulgaris* L. beans and *Leucaena* sp. trees. *Int. J. Syst. Bacteriol.* **1991**, *41*, 417–426. https://doi.org/10.1099/00207713-41-3-417.
2. del Cerro, P.; Pérez-Montaño, F.; Gil-Serrano, A.; López-Baena, F.J.; Megías, M.; Hungria, M.; Ollero, F.J. The *Rhizobium tropici* CIAT 899 NodD2 protein regulates the production of Nod factors under salt stress in a flavonoid independent manner. *Sci. Rep.* **2017**, *7*, 46712. https://doi.org/10.1038/srep46712.
3. Madinabeitia, N.; Bellogín, R.A.; Buendía-Clavería, A.M.; Camacho, M.; Cubo, T.; Espuny, M.R.; Gil-Serrano, A.M.; Lyra, M.C.; Moussaid, A.; Ollero, F.J.; et al. *Sinorhizobium fredii* HH103 has a truncated *nolO* gene due to a -1 frameshift mutation that is conserved among other geographically distant *S. fredii* strains. *Mol. Plant Microbe Interact*. **2002**, *15*, 150–159. https://doi.org/10.1094/mpmi.2002.15.2.150.
4. López-Baena, F.J.; Vinardell, J.M.; Pérez-Montaño, F.; Crespo-Rivas, J.C.; Bellogín, R.A.; Espuny, M.R.; Ollero, F.J. Regulation and symbiotic significance of nodulation outer proteins secretion in *Sinorhizobium fredii* HH103. *Microbiology* **2008**, *154*, 1825–1836. https://doi.org/10.1099/mic.0.2007/016337-0.
5. Acosta-Jurado, S.; Navarro-Gómez, P.; Murdoch, P.S.; Crespo-Rivas, J.C.; Jie, S.; Cuesta-Berrio, L.; Ruiz-Sainz, J.E.; Rodríguez-Carvajal, M.Á.; Vinardell, J.M. Exopolysaccharide production by *Sinorhizobium fredii* HH103 is repressed by genistein in a NodD1-dependent manner. *PLoS ONE* **2016**, *11*, e0160499. https://doi.org/10.1371/journal.pone.0160499.
6. Acosta-Jurado, S.; Alias-Villegas, C.; Navarro-Gómez, P.; Almozara, A.; Rodríguez-Carvajal, M.A.; Medina, C.; Vinardell, J.M. *Sinorhizobium fredii* HH103 *syrM* inactivation affects the expression of a large number of genes, impairs nodulation with soybean and extends the host-range to *Lotus japonicus*. *Environ. Microbiol.* **2020**, *22*, 1104–1124. https://doi.org/10.1111/1462-2920.14897.
7. Vinardell, J.M.; López-Baena, F.J.; Hidalgo, A.; Ollero, F.J.; Bellogín, R.; Espuny, M.R.; Temprano, F.; Romero, F.; Krishnan, H.B.; Pueppke, S.G.; et al. The effect of FITA mutations on the symbiotic properties of *Sinorhizobium fredii* varies in a chromosomal-background-dependent manner. *Arch. Microbiol.* **2004**, *181*, 144–154. https://doi.org/10.1007/s00203-003-0635-3.

8. Figurski, D.H.; Helinski, D.R. Replication of an origin containing derivative of plasmid RK2 dependent on a plasmid function provided in *trans*. *Proc. Natl. Acad. Sci. USA* **1979**, *76*, 1648–1652. https://doi.org/10.1073/pnas.76.4.1648.
